# Accurate primary germ cell cancer diagnosis using serum based microRNA detection (ampTSmiR test)

**DOI:** 10.18632/oncotarget.10867

**Published:** 2016-07-27

**Authors:** Ton van Agthoven, Leendert H.J. Looijenga

**Affiliations:** ^1^ Department of Pathology, Josephine Nefkens Building, Erasmus MC Cancer Institute, Rotterdam, The Netherlands

**Keywords:** testicular germ cell cancer, microRNA, serum biomarker, RT-qPCR, miR-371a-3p/373-3p/367-3p

## Abstract

Multiple studies, including various methods and overall limited numbers of mostly heterogeneous cases, indicate that the level of embryonic stem cell microRNAs (miRs) (e.g. 371a-3p, 372-3p, 373-3p, and 367-3p) are increased in serum at primary diagnosis of almost all testicular germ cell cancer (TGCC).

Here we determine the status of three of these miRs in serum samples of 250 TGCC patients, collected at time of primary diagnosis, compared with 60 non-TGCC patients and 104 male healthy donors. The levels of miRs were measured by the robust ampTSmiR test, including magnetic bead-based miR isolation and target specific pre-amplification followed by real-time quantitative PCR (RT-qPCR) detection. Calibration is performed based on the non-human spike-in ath-miR-159a, and normalization on the endogenous control miR-30b-5p.

The serum levels of miR-371a-3p, 373-3p, and 367-3p are informative to accurately detect TGCC patients, both seminomas and non-seminomas, at the time of primary diagnosis (*p*< 0.000). Receiver Operating Characteristic (ROC) analysis demonstrate that the Area Under the Curve (AUC) for miR-371a-3p is 0.951 (being 0.888 for miR-373-3p and 0.861 for miR-367-3p), with a sensitivity of 90%, and a specificity of 86% (positive predictive value of 94% and negative predictive value of 79%). Inclusion of miR-373-3p and 367-3p resulted in a AUC of 0.962, with a 90% sensitivity and 91% specificity. Similar results were obtained using the raw Ct data. Importantly, the results demonstrate that ampTSmiR is not suitable to detect pure teratoma as well as the precursor of TGCC, i.e., Germ Cell Neoplasia *In Situ* (GCNIS).

The largest series evaluated so far, demonstrate that detection of the embryonic stem cell miR-371a-3p, 373-3p and 367-3p is highly informative to diagnose patients with a primary TGCC.

## INTRODUCTION

Testicular germ cell cancers (TGCC) are rare in the general population, but represent the most common malignancies in Caucasian men aged 20-40 years [1]. TGCC are a clinically and pathologically heterogeneous group of malignancies. TGCC arise from the precursor lesion Germ Cell Neoplasia *In Situ* (GCNIS) [2]. The former nomenclature was carcinoma *in situ* cells, which closely resembles embryonic germ cells, either primordial germ cells or gonocytes [3]. GCNIS can progress into seminomas (SE) and non-seminomas (NS). The NS can consist of the histologic subtypes embryonal carcinoma (EC), yolk sac tumor (YST), teratoma (TE), and choriocarcinoma (CH) [1, 3–7]. Cure rate is over 90% of SE patients (independent of stage), and 100% for low stage I and II disease, and about 70% for stage III and IV disease. For NS patients cure rate is 95% in stage I and II, and approximately 70% for stage III and IV disease [8, 9].

TGCC patients have years or even decades after the initial diagnosis a risk of developing a second TGCC or progression of the disease. Therefore, they require intensive and long term follow-up after diagnosis and treatment. For primary diagnosis and follow-up, various approaches are currently used, including monitoring the diagnostic/follow-up serum-markers α-fetoprotein (AFP), human chorionic gonadotropin subunit beta (hCGB), and to a lesser extent, because of lower specificity, lactate dehydrogenase (LDH) [10]. Approximately 80% of the NS, and 20% of the SE show increased levels of the serum markers. The serum levels of AFP and hCGB are informative to distinguish the good, intermediate and poor risk NS group in the metastatic setting. AFP is primarily related to the presence of a YST component and hCGB to a CH component in the tumor, while they show little sensitivity for the SE and EC components. Therefore, a significant number of TGCC patients without YST and CH, will be negative for these serum markers. In addition false high hCGB levels may be induced by chemotherapy or marijuana use and false AFP levels due to liver damage or disease [10, 11]. Positivity for the markers can also change during disease progression, due to different histology of the metastasis compared with the primary cancer. These shortcomings significantly limit their clinical applicability. Therefore, reliable biomarkers with a better sensitivity are needed, especially markers related to presence of SE and EC.

miRNAs (miRs) are small non-coding RNA molecules of approximately 18–25 nucleotides in length, involved in post-transcriptional gene regulation, by the induction of mRNA degradation or inhibition of protein translation [12]. miRs are involved in several essential biological processes including cell development, differentiation, and diseases. miRs are highly tissue specific and play a key role in differentiation and maintenance of tissue identity. However, the function of most miRs has still to be unraveled. Dysregulation of miRs has been linked to cancer development and progression [13]. In addition, miRs have been identified in many studies as novel diagnostic and prognostic biomarkers and are highly stable in body fluids, including serum, and can survive unfavorable physiological conditions such as multiple freeze–thaw cycles, extended storage, and extreme variations in temperature and pH [14]. In addition, body fluid-based biomarkers are attractive because of the ease and minimal invasiveness of collecting multiple samples to monitor actual disease state.

Earlier research studies identified specific embryonic stem cell miR-371-3 cluster and miR-302a-d/367 cluster as characteristic tissue and serum biomarkers for GCC, including those of the testis [15–18]. The miR-371/3 and miR-302/367 clusters are for sure expressed in SE/EC/YST and are epigenetically silenced in adult somatic cells as well as TE [19–22], most likely irrespective of the anatomic site of the tumor and patient age, with TE as the only exception [16, 17]. CH has not been evaluated separately so far.

In the current study, we examined the levels of miR-371a-3p, 373-3p and miR-367-3p in serum samples of the largest series of TGCC patients at time of primary diagnosis in comparison with non-germ cell cancer (NGCC) patients with other testicular abnormalities and male adult healthy controls. The results demonstrate that miR-371a-3p, 373-3p and 367-3p are significantly elevated in sera of patients with a TGCC. In fact, miR-371a-3p alone is highly informative to diagnose patients with a primary TGCC compared to a healthy control group with a Receiver Operating Characteristic (ROC) Area Under the Curve (AUC) of 0.951, with a sensitivity and specificity of 90 and 86%, respectively. The positive and negative predictive values are 94% and 79%. Combining the three targets results in a AUC of 0.962 and a sensitivity and specificity of 90 and 91%.

## RESULTS

### Comparison of embryonic miR levels between TGCC, NGCC, and healthy individuals

**Figure 1:**
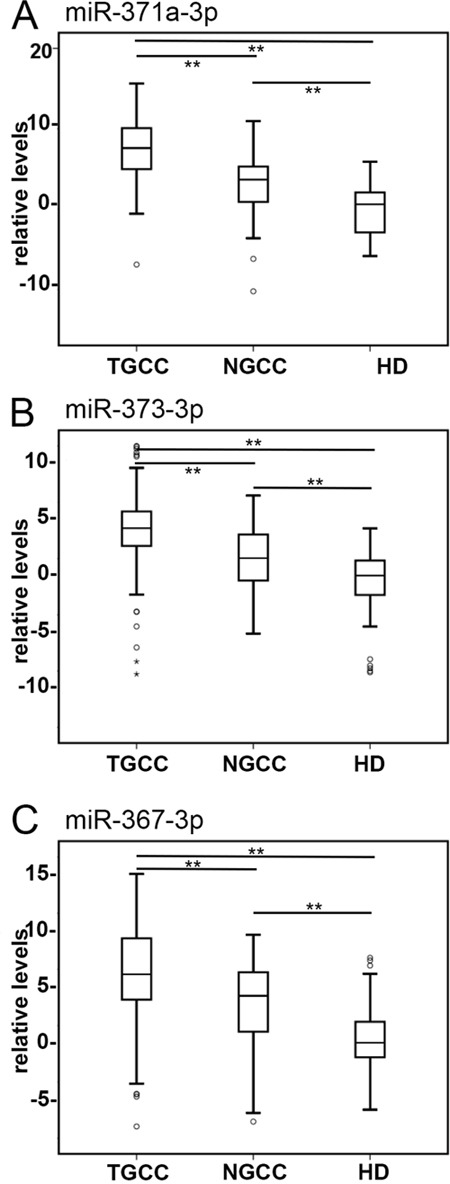
Detection of TGCC by serum levels of tumor associated miRs Boxplots of the relative serum levels is presented of **A**. miR-371a-3p, **B**. 373-3p and **C**. 367-3p of TGCC (n = 238), NGCC (n = 60) at diagnosis, and HD (n =104). Serum levels of **A**. miR-371a-3p, **B**. miR-373-3p, and **C**. miR-367-3p discriminate patients with TGCC from those with NGCC and HD. The box marks the first and third quartiles. Horizontal lines mark median values, circles are outliers, far outliers are indicated with an asterisk. The whiskers are defined as 1.5 times the interquartile range. (**) p<0.000, Mann-Whitney U Test. Abbreviations: TGCC: testicular germ cell cancer; NGCC: non germ cell cancer; HD: healthy donor.

The primary goal of this study was the further optimization of a previously reported detection method for miR in serum (TSmiR test) [15, 23], and to demonstrate the power of the adjusted protocol (ampTSmiR test) for the primary diagnosis of TGCC patients. For this purpose an amplification step and stringent quality check has been included. In brief, the levels of three preselected miRs (miR-371a-3p, miR-373-3p and miR-367-3p) were determined in a RT-qPCR assay after a magnetic bead-based isolation and a targeted pre-amplification step. In spite of some differences in the detection of the spike-in targets (n>400, SD<0.84, coefficients of variation less than 7.1%)(Supplementary Figure S1A), calibration with these spike-ins had minor effects on the levels of the reference miRs (Supplementary Figure S1B) (see Materials and Methods for further details). Therefore, the measured levels were calibrated to the non-human spike-in ath-miR-159a only. The reason for omitting the cel-miR-39-3p as target for calibration, as previously included [15] is to simplify the pipeline, because inclusion of multiple targets for calibration had no additional value (data not shown). Unexpectedly, a significant and relative large difference in detection levels of the reference miR-93 was observed between the TGCC and HD group, compared with miR-30b-5p (Supplementary Figure S1C and S1D). In fact, the HD showed a significant lower level compared with both of the affected groups (GCC and NGCC, p<0.000). This observation could not be made in our previous study [15], because no HD group was included. As a consequence, our previous approach, based on normalization of the median of both miR-30b-5p and miR-93, resulted in a minor reduction on separation of patients and HD compared with the use of miR-30b-5p only (AUC scores improved from 0.937 to 0.951) (data not shown). Therefore in contrast to our earlier publication [15], miR-93 has been excluded for normalization in this study, and the miR levels were normalized to miR-30b-5p, proven to be a reliable stable miR in serum [23]. This protocol, i.e., the ampTSmiR test, was applied to a unique series of sera at the time of primary diagnosis from 250 TGCC patients, 60 NGCC patients and 104 HD. The overlap of cases with the earlier study [15] is specified in the Materials & Methods section. Comparison of the analyses indicated similar results but a higher specificity, which shows the additional value of the improved protocol regarding calibration, normalization and the additional use of an amplification step in the pipeline. The miR levels from the cohort of 250 sera from TGCC patients, including 110 NS, 128 SE, six TE and six GCNIS, 60 NGCC patients and 104 male HD were compared. Box plots of relative levels of circulating miRs are depicted in Figure 1 and in principal component analysis (PCA) plots (Supplementary Figure S2). A clear separation of TGCC and HD by levels of miR-371a-3p, 373-3p and 367-3p was observed (each *p* < 0.000). NGCC and HD were significantly separated by levels of miR-371a-3p, 367-3p, and by miR-373-3p as well (*p* < 0.000). Importantly, sera of TGCC and NGCC patients were separated by levels of the three miRs (each *p* < 0.000) (Figure 1). As expected, the highest levels of the miRs were observed in sera of TGCC patients (Figure 1). A positive relation was observed between tumor size and levels of miR-371a-3p in patients with known stadium I TGCC, both SE and NS (n = 72, Pearson correlation 0.429, *p*<0.000), in agreement with a recent study [24] (Supplementary Table 1). Also a relation was observed for levels of miR-373-3p and miR-367-3p and tumor size in patients with stadium I TGCC. This correlation was lost in case of proven metastatic disease, for the total group as well for the SE and NS patients specifically.

### Diagnostic information of the serum based miR-371a-3p, 373-3p and 367-3p were determined by receiver operating characteristic (ROC) analysis

**Figure 2:**
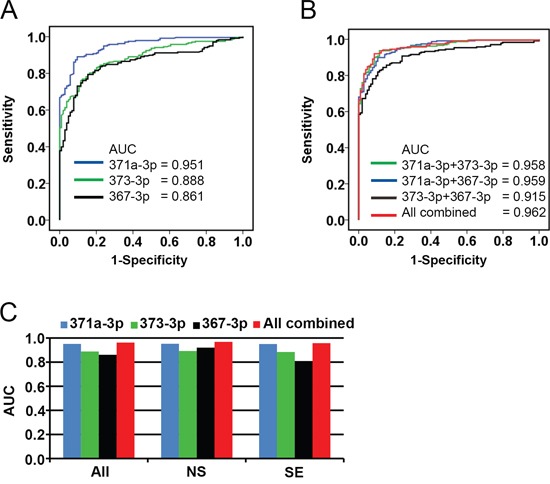
Receiver operating characteristic (ROC) plot **A**. Diagnostic accuracy of circulating miR-371a-3p, 373-3p and 367-3p were analyzed by ROC curves. The data shown in Figure 1 were used to draw the ROC plots. The Area Under the Curve (AUC) varied from 0.861 to 0.951. **B**. ROC curves generated of combinations of the different miR analyses. The AUC varied from 0.915 to 0.962 for combination of the miRs. **C**. ROC analysis generated separately for sera of SE and NS patients. The AUC varied from 0.920 to 0.968 for NS and from 0.809 to 0.957 for SE.

**Figure 3:**
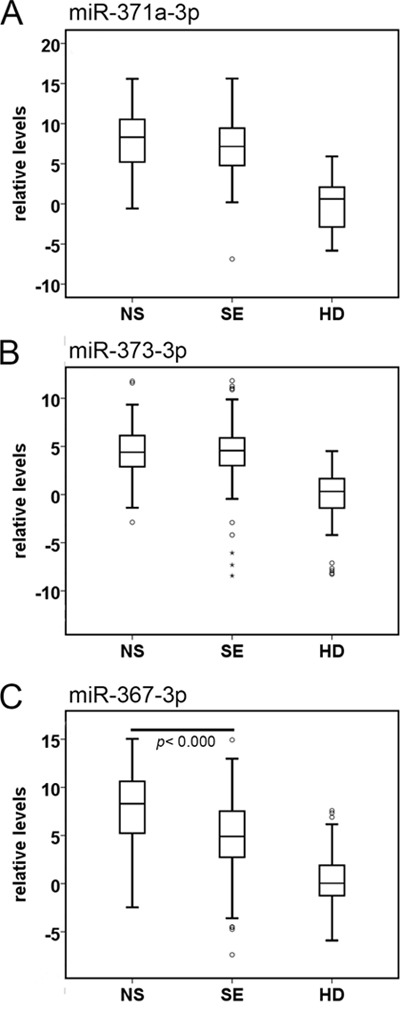
Boxplots of the relative serum levels **A**. miR-371a-3p, **B**. miR-373-3p, and **C**. miR-367-3p of NS (*n* = 110), SE (*n* = 128), and HD (*n* =104). For comparison of the levels of each miR in serum of NS or SE with HD: *p*<0.000, Mann-Whitney U Test. Abbreviations: NS: non-seminoma; SE: seminoma; HD: healthy donor.

A ROC curve was generated for each miR separately (Figure 2A) and the various combinations of the miRs (Figure 2B). Area under the curve (AUC) for miR-371a-3p was 0.951 for the comparison of TGCC with HD, being 0.888 for miR-373-3p and 0.861 for miR-367-3p. The list of subgroups with confidence intervals is indicated in Supplementary Table S2, and partly represented in Figure 2. Combination of miR-371a-3p, 373-3p and 367-3p resulted in a AUC of 0.962. Separation of TGCC in NS and SE cases gave similar results with slightly higher AUC values for NS patients for the individual targets, most likely caused by higher levels in NS serum samples compared with sera of SE patients (Figure 2C and 3). Levels of miR-367-3p were significantly higher in NS compared with SE serum samples (*p* <0.000). Of notice is that the RAW data (i.e., without calibration or normalization) gave the similar significant separation of TGCC patients and HD based on the individual targets (Supplementary Figure S3)

Cut-off points were calculated with the optimal balance between sensitivity and specificity (Supplementary Table S2, indicated with an asterisk). For miR-371a-3p comparison of TGCC with HD resulted in a sensitivity of 89% and a specificity of 90%. For miR-373-3p a sensitivity of 70% and specificity of 89% was reached. For miR-367-3p the sensitivity was 79%, with a specificity of 85%. The combination of all three miRs resulted in a sensitivity of 92% and a specificity of 91%. Inclusion or exclusion of specific targets besides miR-371a-3p has minor effect. Sensitivity and specificity were also calculated when set at 90% (Supplementary Table S2). Positive predictive value (PPV) calculated for the best discriminating miR, being miR-371a-3p, was 94% and a negative predictive value (NPV) of 79% with a sensitivity of 90 and specificity of 86%.

### Comparison of circulating miR levels between GCNIS, TE patients and healthy individuals

**Figure 4:**
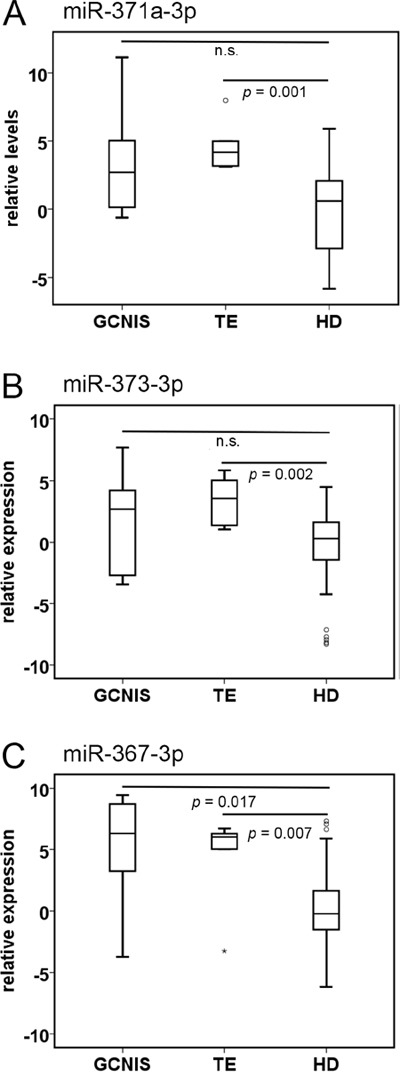
miR levels in individuals with pure Germ Cell Neoplasia *in situ* (GCNIS), or teratoma (TE) Boxplots of the relative serum levels of **A**. miR-371a-3p, **B**. 373-3p and **C**. 367-3p of patients with GCNIS (*n* = 6), TE (*n* = 6), and HD (*n* =104). *p-*values, Mann-Whitney U Test. Abbreviations: n.s. non-significant.

This random selected series of sera contained six cases of GCNIS only (i.e., no invasive component identified) and six cases of pure mature TE. GCNIS showed similar levels of miR-371a-3p and 373-3p and higher levels of miR-367-3p (*p* = 0.017) compared with HD (Figure 4). TE related samples showed higher levels of miR-371a-3p (*p* = 0.001), miR-373-3p (*p* = 0.002) and miR-367-3p (*p* = 0.007) than HD (Figure 4). Although the number of cases investigated is low, the results already indicate that it is unlikely that GCNIS or TE only can be diagnosed based on the ampTSmiR test, due to the small differences of the miR levels. This is further strengthened by a single case diagnosed as GCNIS only in the testis, based on pathological classification, although the matched serum showed elevated levels of all three miRs. Review of the clinical data, indicated lung and para aortic lymph node metastases with high levels of hCGB, supportive for a metastatic TGCC. This case, based on these data, was therefore included in the TGCC cohort investigated.

### Comparison of serum miR levels between NGCC and TGCC

**Figure 5:**
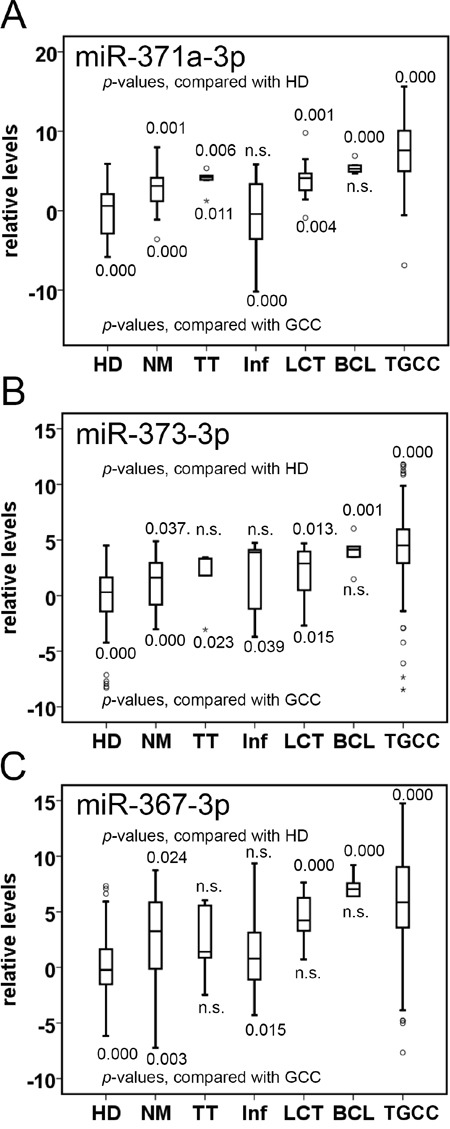
miR levels in individuals with testicular disease Boxplots of the serum levels of **A**. miR-371a-3p, **B**. miR-373-3p, and **C**. miR-367-3p of NGCC subgroups, HD (*n* =104), and TGCC (GCC) patients (n = 236). Abbreviations: NM, no malignancy (n = 20); TT, torsio testis (n = 8); Inf, inflammation (n = 12); LCT, Leydig cell tumor (n = 8); BCL, B-cell lymphoma (n = 5); n.s. non-significant. *p-*values, Mann-Whitney U Test.

The series contained 60 sera of a heterogeneous group of NGCC cases. Within this group, the testes were removed or biopsied for various reasons, in most cases suspicion of a testicular cancer. As shown in Figure 1, Supplementary Figure S1B, and Supplementary Table S2 separation of TGCC and NGCC is less perfect than separation of TGCC and HD. To address the question whether this was caused by a specific subgroup, boxplots were generated and statistical analysis was performed (Figure 5). Although the number of samples in the different subgroups is relatively small, some subgroups show significantly different results compared with the samples of the HD. For example, higher levels of miR-371a-3p were found in cases with no malignancy (NM), torsio testis (TT), Leydig cell tumor (LCT), and B-cell lymphoma (BCL). miR-373-3p was elevated in NM, LCT, and BCL only. miR-367-3p was elevated in cases with NM, LCT, and BCL. For the comparison of the subgroups with TGCC, the five cases with BCL showed similar levels of each miR. No differences related to the normalization target(s) were however identified (see above).

## DISCUSSION

miRs detected in serum have been described as informative for both diagnosis and prognosis of TGCC. Various small scale studies have been performed using different approaches and heterogeneous patient cohorts. The first case report of Murray *et al*. [25] used conventional RNA isolation in combination with multiplexed, high throughput cDNA synthesis and quantification of serum miRs. All eight main miRs of the miR-371/3 and miR-302/367 clusters were highly elevated in the serum of a four-year-old boy at the time of diagnosis of a malignant YST. Levels fell during chemotherapy and returned to normal during an uneventful clinical follow-up. Another study [26] investigated sera of six SE and five NS patients with stage I disease before orchiectomy and five days post-operative and compared with sera of twelve healthy males. The pre-operative serum levels of the miRs 371a-3p, 372-3p and 373-3p were found to be higher than the levels detected in controls, and all declined after treatment. In addition, only two of the patients included had elevated levels of the classical serum markers. In two studies by Dieckmann and Spiekermann *et al*. [27, 28] the miRs of the miR-371/3 cluster were quantified in serum samples of 24 TGCC patients before and after treatment. In all patients, serum levels of miRs 371-3-3p were elevated compared to controls and dropped after orchiectomy. In cases with advanced disease levels dropped to the normal range after completion of treatment. The levels of miR-371a-3p were not correlated with the levels determined in the primary cancer tissue. This is in line with a previous study from our group [22].

Another study from our group provided evidence that the serum levels of the selected embryonic specific miRs is informative to detect a TGCC in the majority of cases for initial diagnosis, outperforming the classical protein serum markers hCGB and AFP [15]. A miR-specific para-magnetic bead capture system was used to isolate and purify miRs from serum with subsequent RT-qPCR. The Targeted serum miR (TSmiR) test was applied to five independent serum sample series including 80 TGCC, 47 controls, 11 matched pre- and post-orchidectomy samples and 12 NGCC testicular masses. These cases had overlap with the previous studies from the UK and Germany, showing similar observations, supporting the informativity of the TSmiR test. TGCC serum samples showed a significant increase of miR-371-3/367-3p levels. miR serum levels returned to normal levels after orchidectomy (similar cases as reported before [26]). Furthermore, there was a trend toward higher miR levels in patients with disseminated disease. The TSmiR test showed an overall sensitivity of 98%, and a specificity of 48% (control versus cancer), based on combination of the different miRs, outperforming the serum markers AFP and hCGB. Also Syring *et al*. [29] reported that miR-371a-3p outperformed AFP and hCGB in detection of TGCC. Recently a robust pipeline was described, using total RNA isolation and pre-amplification, for detection of miR-371-3p and 367-3p in sera and cerebrospinal fluid of 25 pediatric GCC patients [23]. The method showed high sensitivity and specificity for the diagnosis of extracranial GCC. It allowed early detection of relapse in one patient with a GCC and distinguished intracranial GCC from intracranial non-GCC at diagnosis.

So far, in total more than 130 serum samples of TGCC and over 70 controls have been evaluated, although with different protocols. This makes a proper conclusion regarding the value of the test difficult, especially in the context of the used heterogeneous control samples. Therefore, amongst others, the present study was undertaken, including the largest series of sera from primary TGCC patients (i.e., n = 250), including both SE and NS, were compared with sera from male HD (n = 104) and sera from NGCC (n = 60). In addition to the miR-specific paramagnetic bead capture, pre-amplification of the cDNA was added to the pipe-line to increase sensitivity and specificity. The results demonstrate that sera from both NS and SE could be separated with high sensitivity and specificity from sera of HD by levels of miR-371a-3p, 373-3p and 367-3p (Supplementary Table S2). This means that the ampTSmiR test is an important new tool in the clinical management of TGCC. A possible shortcoming of the study could be the difference in age between the subgroups, especially the additional healthy control group, being healthy blood donors (HD) (see Materials & Methods section for more details). However, no relation between age and miR levels in the different subgroups was identified, and therefore concluded that the HD is a relevant and informative subgroup in the context of the study performed.

Interestingly, we observed higher levels of the selected embryonic miRs in some subgroups of NGCC but with other testis abnormalities. This is in line with a relatively low level of these miRs in normal testis, as we reported before [17]. However, the mechanisms resulting in the release of these miRs is unknown. Several possibilities have been suggested, including passive leakage from damaged cells, due to tissue injury, chronic inflammation, necrosis or active secretion via micro-vesicles, including exosomes [14]. In this context, it is of notion that in our series no increased miR levels were observed in cases with inflammation or necrosis, including torsio testis for miR-373-3p and miR-367-3p. In contrast, in cases of Leydig cell tumor and B-cell lymphoma increased levels of miR-371a-3p, 373-3p and 367-3p were observed in serum. To rule out that this was caused by normalization, a boxplot was generated showing the levels of miR-30b-5p in sera of the various patients, i.e., TGCC, HD, and NGCC split in malignancy and no malignancy (Supplementary Figure 1E). In spite of presence of a minor difference between HD and TGCC (0.6 Ct), and HD and NGCC (0.9 Ct) (Figure S2C), no significant difference was observed between the NGCC group split in malignant and non-malignant. The increased miR levels may give a problem in differential diagnosis under certain conditions, therefore biopsy remains the golden standard in suspected cases, like in case of a mono-testis. In that particular situation the direct enzymatic alkaline phosphatase reactivity staining might be informative to be included to prevent a second operation [30]. Although it needs further investigation, our preferred hypothesis is that the elevated levels are explained by a disturbed integrity of the testis. Cellular stress might be the cause of the elevated serum miR levels in these patients with testicular complaints. The group with inflammation contained one patient with inflammation of the testis and the others suffered from epididymitis, which did not disturb the integrity of the testis. This finding must be kept in mind in the context of the use of these miR for primary diagnosis of a TGCC.

Several risk factors for development of TGCC have been identified. Both high and low birth weight, cryptorchidism, inguinal hernia, twinning, and gestational age are factors influencing the risk of development of a TGCC [31]. Also patients with Disorders of Sex Development are at risk for gonadal, including TGCC [32]. It would be most useful to identify patients at a very early stage of the disease (GCNIS) within the groups with an increased risk using a non-invasive test.

So far, all results indicate that the selected miRs are present in seminoma and all the different histological elements of non-seminoma (except teratoma), i.e., embryonal carcinoma, yolk sac tumor and choriocarcinoma [15, 23] and unpublished observations. The data presented here indicate that the ampTSmiR assay as performed will not be able to detect the single presence of the precursor lesion GCNIS as well as pure teratoma both at primary diagnosis as well as during follow up. The first entity is of relevance, because it will allow identification of patients with the presence of invasive disease, independent of the histological composition, except pure teratoma, both seminoma as well as (mixed) non-seminoma. This is nicely demonstrated by the single patient with elevated miRNA levels and only GCNIS at pathological examination of the suspected testis, but evidence of metastatic disease with lung and para-aortic lymph node metastases and raised levels of HCG, consistent with a malignant GCT. The putative second limitation of the assay, i.e., failure to detect pure TE in contrast to the non-teratomatous non-seminomatous elements (EC, YST, CH), is of importance for the role of the ampTSmiR assay in a clinical setting as well. The inability to detect pure TE compared to the other nonseminomatous elements might be highly beneficial of the test in daily practice. High throughput miR profiling experiments are currently ongoing to identify TE specific informative miRNA targets, although it is unlikely that these will be found based on the pure somatic differentiation. In other words, additional biomarkers will be required for this specific entity.

We observed a significant correlation between the level of the target miRNAs and the size of the primary lesion in stage I disease, both seminoma and non-seminoma, independent of histological composition. This correlation is lost in case of metastatic disease both for seminoma and non-seminoma. Further investigation of this finding, in line with an independent recent publication [24], might result in the level of serum miRNA as predictor of the presence of metastatic disease in case the size of the primary GCC is known. This is currently under investigation. Moreover, it will be of relevance in case of marker negative GCC, found to be the case in 20% of the NS and 80% of the SE patients. It is not expected that, besides TE, seminoma or the other histological component of non-seminoma will become negative for these miRNA upon further clinical progression. This is based on the intrinsic characteristics of these markers for the embryonic status of the GCC (non-TE) elements. In addition, pure TE are, so far, found to be overall negative for the miRs as identified. In this study moderately elevated levels were however found, likely due to the increased sensitivity of the assay. The impact of detection of pure TE in a clinical setting requires further investigations, which are currently ongoing. The overall negative pattern is expected, based on loss of the embryonic characteristics of this specifically and completely somatically differentiated histology, to be independent of anatomic localization. This fact might be informative in the clinical context of prediction of a non-TE (malignant) component in metastases, both under active surveillance as well as systemic treatment (either irradiation of chemotherapy). With a single exception [23], no further data is available related to growing teratoma, as well as non-germ cell malignant progression, which is a topic of current investigation. Moreover, as briefly indicated above, the ampTSmiR test might also be highly informative in the context of extragonadal GCC, again of the various histological compositions, likely except pure TE. Supportive data for this assumption has been recently published, indicating that the target miR to be determined whether in serum or cerebrospinal fluid is informative for intracranial GCC as well [23]. To answer these clinically highly relevant questions, it will be necessary to perform additional, preferentially multicenter, both retrospective as well as prospective studies. Therefore, comparative analyses of the profile of the miRs and the standard serum biomarkers (at least AFP and hCGB) as well as clinical data need to be included to decide upon the additional value of the ampTSmiR assay for both the initial diagnosis and follow up. For this it is of relevance that the ampTSmiR pipeline is composed of a defined set of commercially available components. Therefore it is expected that implementation of the ampTSmiR pipeline will be possible for diagnostic departments with expertise on (molecular) biomarker assays in body fluids.

In conclusion, we demonstrate that the serum-based ampTSmiR test is highly sensitive and specific in the identification of TGCC patients compared to HD and patients with non-cancerous testicular diseases. Our next step in this analysis is identification of recurrent or relapsed disease, for which the collected samples, both retrospectively as well as prospectively, are currently under evaluation.

## MATERIALS AND METHODS

### Clinical samples

The study is approved by the institution's Medical Ethical Committee (MEC 02.981 and CCR2041). We adhere to the “Code for Proper Secondary Use of Human Tissue in The Netherlands” developed by the Dutch Federation of Medical Scientific Societies (FMWV (Version 2002, update 2011). Serum samples were collected between January 2003 and July 2015, and stored after primary handling at -80 °C. In total, 250 serum samples of patients with a primary TGCC ((128 SE, median age was 36 (range 21-72 years), 110 NS, median age 29 (range 17-63 years), six TE (median age 29.5 (range 16-65 years), and six GCNIS only (median age 24.5 (range 12-51 years)) were included. miR levels of TGCC patients were compared with 104 sera of male healthy donors, median age 54 (range 26-69 years), and 60 sera of patients with various non-TGCC abnormalities of the testis (median age 44 (range 13-81 years). The latter group consisted of the following variants: NM (no malignancy; n = 20); TT (torsio testis; n = 8); Inf (inflammation; n = 12); LCT (Leydig cell tumor; n = 8); BCL (B-cell lymphoma; n = 5). The normal control serum samples were obtained from Sanquin (Amsterdam, the Netherlands). In total 66 of the 414 serum samples overlap with our earlier presented study [15].

### miRNA purification and quantitative real-time PCR (RT-qPCR)

Specific miRs were isolated from 50 μl serum using target specific anti-miR magnetic beads as described before [15], with the following modifications. A MagMax™ Express-96 robot with TaqMan® miRNA ABC Purification Kits, panel A, was used to isolate the miRs, as per manufacturer's instructions (Thermo Fisher Scientific, Bleiswijk, the Netherlands). cDNA generation and quantification of miR levels was performed using the various TaqMan MicroRNA assays (Thermo Fisher Scientific): catalog ID: hsa-miR-371a-3p (002124); hsa-miR-373-3p (000561); has-miR-367-3p (000555); ath-miR-159a (000338); cel-miR-39-3p (000200); hsa-miR-93 (000432); hsa-miR-30b-5p (000602). In brief 5 μL of specifically targeted purified miR in elution buffer was reverse transcribed into miR specific cDNA with a TAQMAN(R) MICRORNA RT KIT (Thermo Fisher Scientific), followed by a 12 cycle pre-amplification step using 2x TaqMan Preamp Master mix (4488593, detailed protocol by supplier Thermo Fisher Scientific) and 20x TaqMan MicroRNA Assays. Thermal-cycling conditions: 95 °C for 10 min., followed by 12 cycles of 95 °C for 15 sec. and 60 °C for 1 min. miR levels were determined in1.5 μl of cDNA on a TaqMan 7500 Real-Time PCR system, according to the supplier (Thermo Fisher Scientific).

### Quality control

All sera were visually inspected and hemolytic samples were discarded from analysis. Two non-human miR spike-ins ath-miR-159a and cel-miR-39-3p were added in the same fixed amount to the sera (0.2 ul of a 1 nM stock solution) for quality control of RNA isolation and cDNA generation. The fact that equal quantities of ath-miR-159a and cel-miR-39-3p spike-ins do not give identical Ct values is not surprising because of multiple reasons. These include the production of identical concentrations of two different RNA oligo's (with possible differences in quality and requiring over 10,000-fold dilution of the original stock) being by definition subject to variation; in any quantitative PCR analysis, comparison of Ct values (and as such derived levels) between different targets is not directly informative because of potential differences in efficiencies of both the RT-reaction and the actual amplification (PCR). The absolute Ct values for these spike-ins is therefore of no importance, but, they should be recovered and detected stably and reproducibly. Based on the data presented in the manuscript this is the case, since the variation in Ct for these spike-ins is generally within +/- 1 Ct of the average (N>400, SD<0.84 for both, Coefficients of variation are less than 7.1%). Pearson correlation between the results obtained from these spike-ins is 0.367. The relative low correlation is explained by the very small window of variation. Therefore, for calibration of input levels ath-miR-159a was used in order to correct for possible differences in efficiency of the RNA extraction and cDNA generation (Supplementary Figure S1A). No samples had to be excluded due to poor miRNA recovery. For normalization the mean levels of the endogenous reference miR (miR-30b-5p) was used as described before [15, 23]. The miR-93 was omitted for reasons indicated in the Results section. Relative expression is depicted as median dCt of all HD minus dCt miR of interest. In each cDNA synthesis experiment, 10-fold dilution series of purified miR of the TCam-2 seminoma cell line was included for quality control and qPCR efficiency and interplate calibration. For negative control, the no template control, elution buffer was added instead of purified miR.

### Statistical analysis

Data processing and statistical analysis was performed using Microsoft Excel 2010, IBM SPSS statistics V21.0, and GenEx 6 (MultiD). Inter-group differences were compared using the Mann-Whitney U test. A *p* value of <0.05 was considered statistically significant. All *p* values are two-tailed. Receiver operating characteristic (ROC) curves were generated to calculate the area under the curve (AUC), with 95% confidence intervals (CI), and the diagnostic value including sensitivity and specificity. The estimated functions of combined markers were constructed using binary logistic regression. The optimum cut-off value for diagnosis was achieved by maximizing the sum of sensitivity and specificity, (square root of [1-sensitivity]^2^ + [1-specificity]^2^), by minimizing the distance of the cut-off value from the top-left corner of the ROC curve and the maximum distance to the random chance line (Youden index).

## SUPPLEMENTARY MATERIALS FIGURES AND TABLES


