# Supplementary material for: Accurate primary germ cell cancer diagnosis using serum based microRNA detection (ampTSmiR test)
Source: Oncotarget. 2016 Jul 27;8(35):58037–49. doi: 10.18632/oncotarget.10867 (PMC5601631; doi:10.18632/oncotarget.10867)
Supplement: Supplementary file 1 [file oncotarget-08-58037-s001.pdf]

Accurate primary germ cell cancer diagnosis using serum based microRNA detection (ampTSmiR test)

SUPPLEMENTARY FIGURES AND TABLES

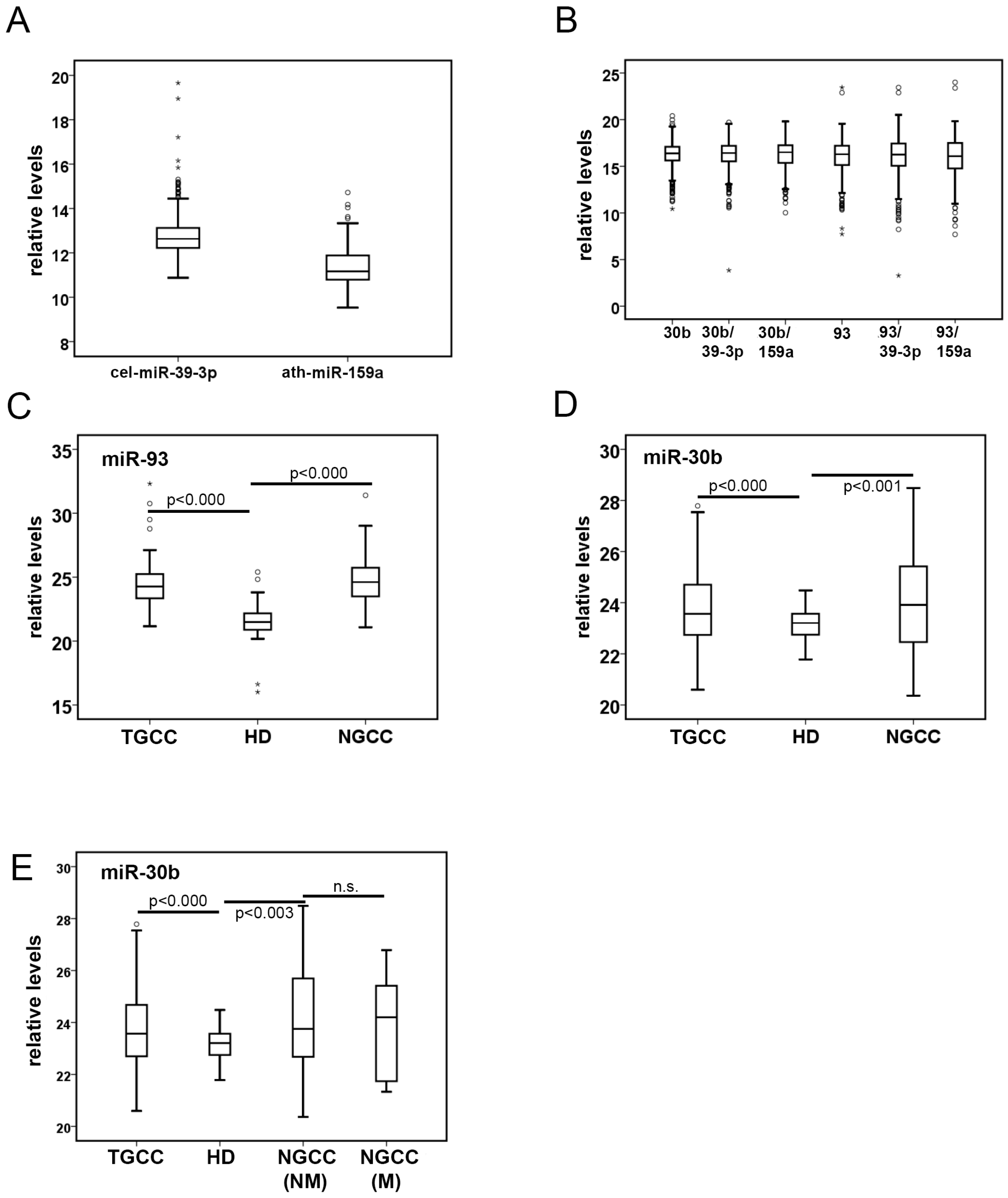

Supplementary Figure S1: A. Variability of the recovery of the spike-ins is presented. The median values are 12.7, and 11.4, SD < 0.84. Coefficients of variation are less than 7.1%. **B.** Effect of calibration with cel-miR-39-3p or ath-miR-159a on raw levels of reference miR-30b and miR-93. **C.** Boxplots of levels of miR-93 in sera of TGCC, HD and NGCC. **D.** Boxplots of levels of miR-30b-5p in sera of TGCC, HD and NGCC. Levels of miR-30b-5p were used to normalize the levels of miR-371a-3p, miR-373-3p, and miR-367-3p. **E.** Levels of miR-30b-5p in sera of patients with TGCC, HD, NGCC without a malignancy (NM) and with a malignancy (M).

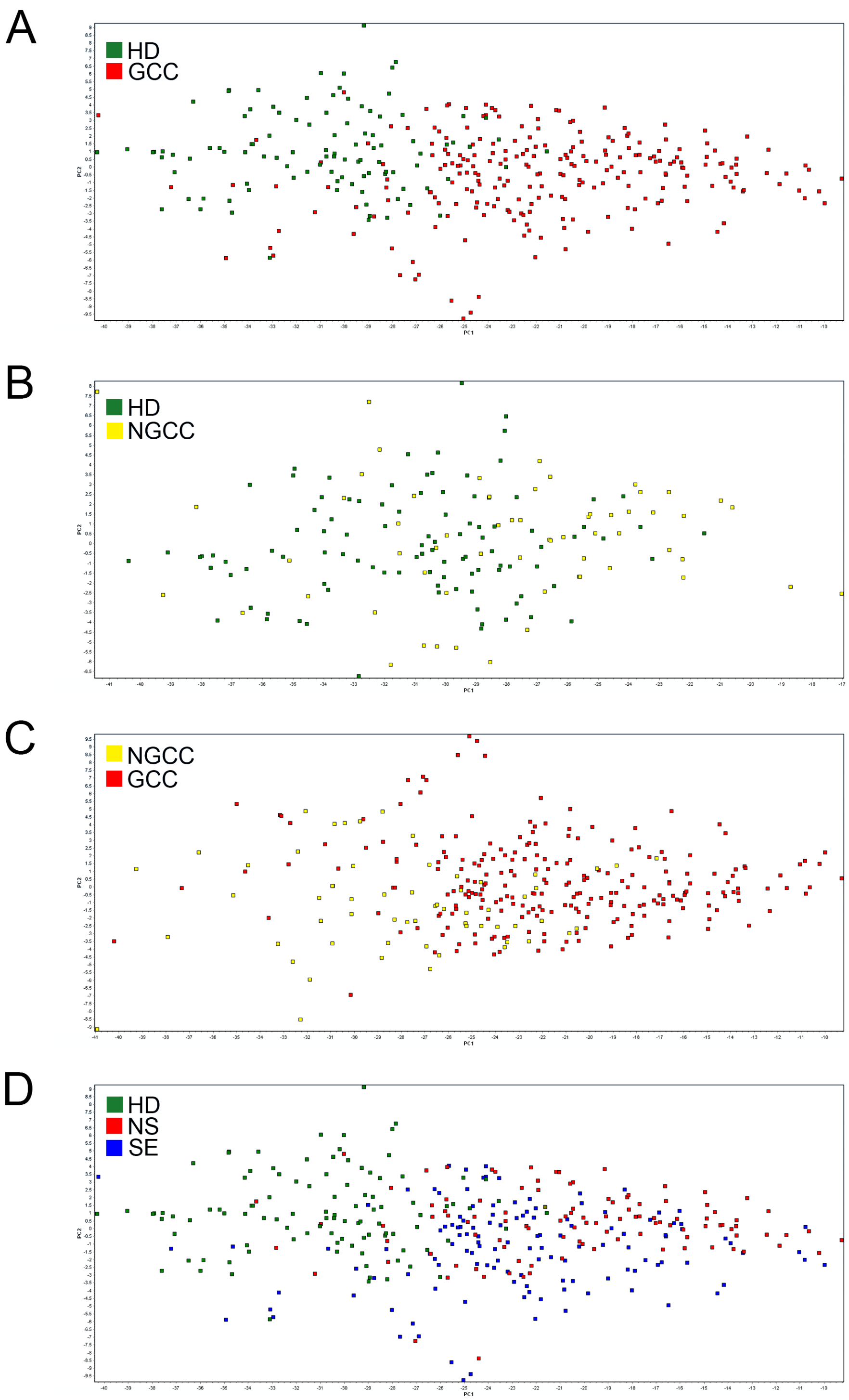

Supplementary Figure S2: A. Principal component analysis (PCA) of miR371a-3p, 373-3p and 367-3p in serum samples of germ cell cancer (GCC) patients and healthy donors (HD). **B.** PCA of non-germ cell cancer (NGCC) patients and HD. **C.** PCA of GCC and NGCC patients. **D.** PCA of non-seminoma (NS), seminoma (SE) and HD.

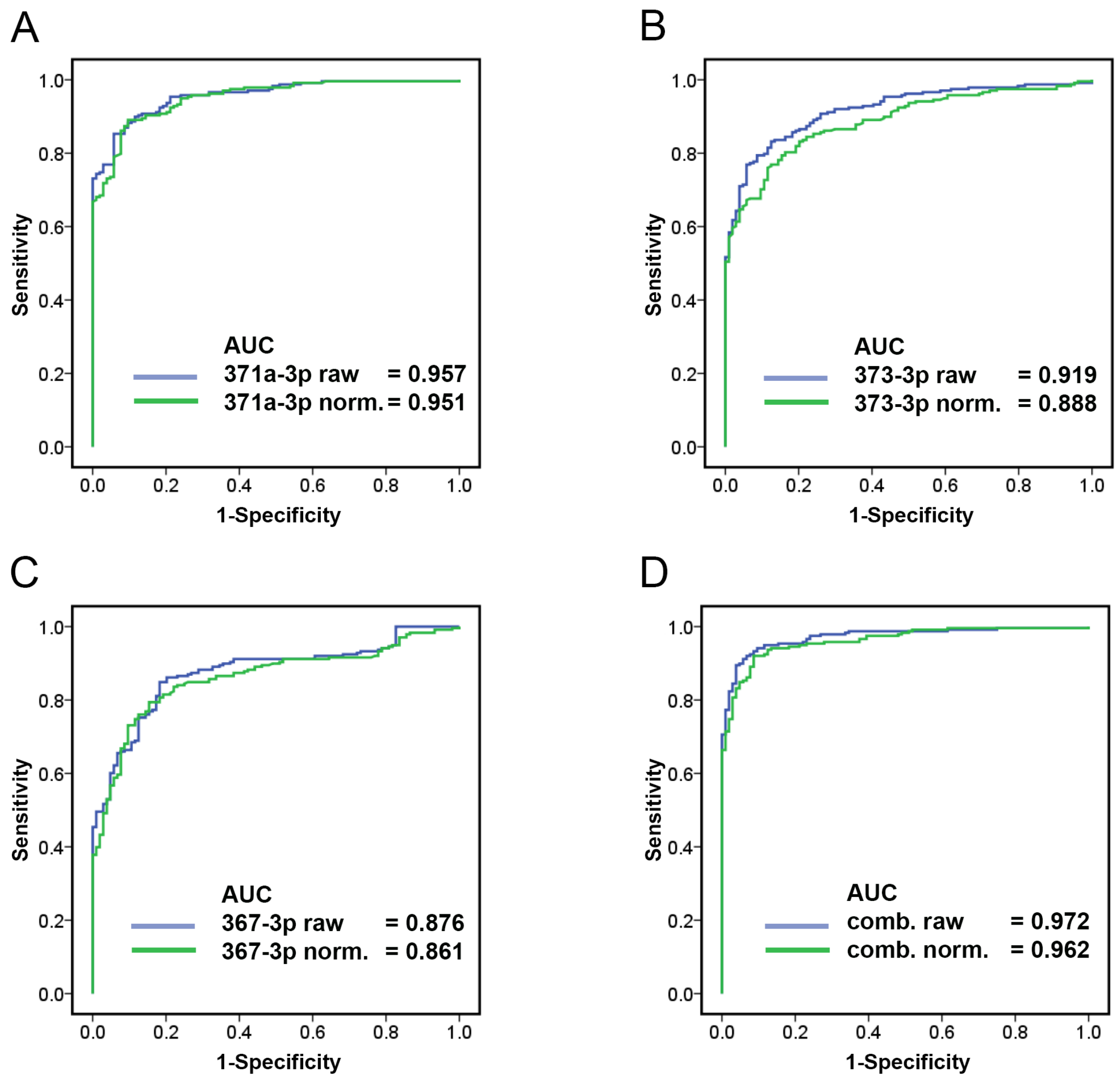

Supplementary Figure S3: Receiver operating characteristic (ROC) plot. Diagnostic accuracy of circulating miR-371a-3p **A.** miR-373-3p **B.** miR-367-3p **C.** and combined miR levels (C). Presented: raw data (raw), or calibrated with levels of spike-in miR-159 and normalized with levels of miR-30b (norm.). Area under the curve (AUC) of the combined raw data is 0.972. Sensitivity 90%, specificity 94.2%.

Supplementary Table S1: Pearson correlation and p values of tumor sizes and miR levels in sera from patients with known and unknown tumor stage

Tumor size	No.	miR-371a-3p	miR-373-3p	miR-367-3p
All	224	0.254, p=0.000	0.198, p=0.003	0.142, p=0.034
Known stage	104	0.233, p=0.017	0.224, p=0.022	n.s.
Stage I	72	0.429, p=0.000	0.246, p=0.037	0.280, p=0.017
NS	34	0.455, p=0.007	n.s.	n.s.
SE	38	0.404, p=0.012	0.350, p=0.031	n.s.
Stage > I	32	n.s.	n.s.	n.s.

No. number of patients, n.s. not significant.

Supplementary Table S2: Diagnostic efficacies of miR-371a-3p, miR-373-3p and miR-367-3p

Comparisons	No. patients	AUC (95% CI)	Sens.%	Spec.%	PPV %	NPV %
**TGCC vs. HD**	**238/104**					
			89	90		
**371a-3p**		0.951 (0.930-0.972)	90	86	94	79
			89*	90	95	77
			70	90		
**373-3p**		0.888 (0.854-0.922)	90	57	78	64
			70*	89	94	58
			73	90		
**367-3p**		0.861 (0.821-0.900)	90	49	81	67
			79*	85	92	63
			92	90		
**All combined**		0.962 (0.943-0.980)	90	91		
			92*	91		
**NS vs. HD**	**110/104**					
			89	90		
**371a-3p**		0.952 (0.927-0.978)	90	86	86	91
			89*	90	90	90
			71	90		
**373-3p**		0.892 (0.850-0.935)	90	63	70	87
			80*	86	88	81
			84	90		
**367-3p**		0.920 (0.883-0.957)	90	78	78	86
			84*	90	89	85
			94	90		
**All combined**		0.968 (0.947-0.988)	90	92		
			94*	91		
**SE vs. HD**	**128/104**					
			89	90		
**371a-3p**		0.950 (0.924-0.966)	90	82	89	87
			89*	90	92	85
			69	90		
**373-3p**		0.884 (0.841-0.927)	90	55	73	80
			84*	80	85	78
			64	90		
**367-3p**		0.809 (0.752-0.866)	90	22	60	64
			72*	85	86	70
			91	90		
**All combined**		0.957 (0.931-0.982)	90	91		
			91*	91		

(*Continued*)

**TGCC vs NGCC**	**238/60**					
**371a-3p**		0.811 (0.755-0.867)	55<br>90<br>68*	90<br>43<br>82	86<br>94	53<br>40
**373-3p**		0.751 (0.687-0.814)	48<br>90<br>57*	90<br>33<br>84	85<br>93	51<br>30
**367-3p**		0.678 (0.611-0.746)	37<br>90<br>36*	90<br>20<br>95	82<br>97	32<br>27
**All combined**		0.804 (0.746-0.862)	53<br>90<br>78*	90<br>43<br>67		

Supplemental Table S2. Diagnostic efficacies of the individual miR-371a-3p, 373-3p and 367-3p as well as the combinations. AUC with 95% confidence interval is presented. ROC analysis was used to determine sensitivity and specificity. The optimal cut-off value for diagnosis is presented with an asterisk (*). Positive predictive value (PPV) and negative predictive value (NPV) is presented at 90% sensitivity and at optimal cut-off (*). Abbreviations: AUC, area under the curve; CI, confidence interval; Sens., sensitivity; Spec., specificity; PPV, positive predictive value; NPV, negative predictive value; TGCC, testicular germ cell cancer; vs, versus; HD, healthy donor; NS, non-seminoma; SE, seminoma; NGCC, non-germ cell cancer.
